# Gesture–Speech Integration in Typical and Atypical Adolescent Readers

**DOI:** 10.3389/fpsyg.2022.890962

**Published:** 2022-06-03

**Authors:** Ru Yao, Connie Qun Guan, Elaine R. Smolen, Brian MacWhinney, Wanjin Meng, Laura M. Morett

**Affiliations:** ^1^China National Institute of Education Sciences, Beijing, China; ^2^School of Foreign Studies, Beijing Language and Culture University, Beijing, China; ^3^Teachers College, Columbia University, New York, NY, United States; ^4^Department of Psychology, Carnegie Mellon University, Pittsburgh, PA, United States; ^5^Department of Moral, Psychological and Special Education, China National Institute of Education Sciences, Beijing, China; ^6^Department of Educational Studies in Psychology, Research Methodology, and Counseling, University of Alabama, Tuscaloosa, AL, United States

**Keywords:** deaf and hard of hearing, automaticity, gesture–speech integration, spoken language comprehension, gesture

## Abstract

This study investigated gesture–speech integration (GSI) among adolescents who are deaf or hard of hearing (DHH) and those with typical hearing. Thirty-eight adolescents (19 with hearing loss) performed a Stroop-like task in which they watched 120 short video clips of gestures and actions twice at random. Participants were asked to press one button if the visual content of the speaker’s movements was related to a written word and to press another button if it was unrelated to a written word while accuracy rates and response times were recorded. We found stronger GSI effects among DHH participants than hearing participants. The semantic congruency effect was significantly larger in DHH participants than in hearing participants, and results of our experiments indicated a significantly larger gender congruency effect in DHH participants as compared to hearing participants. Results of this study shed light on GSI among DHH individuals and suggest future avenues for research examining the impact of gesture on language processing and communication in this population.

## Introduction

With the diagnosis of hearing loss increasing in prevalence (Yuri et al., 2008; Verrecchia and Curcio, 2016) and being researched around the world (Moscicki et al., 1985; Cruickshanks et al., 1998; Reuben et al., 1998; Colozza and Anastasio, 2009), experts are debating an important question: does the loss of hearing mean simply the absence of sensory input in the auditory modality, or does it lead to enhancement of perceptual ability in other modalities, such as vision? Compensatory plasticity holds that the lack of auditory stimulation experienced by deaf individuals is accompanied by enhancements in other senses, such as visual cognition. However, some evidence in the educational and cochlear implant literature documents deficient visual cognition in individuals who are deaf or hard of hearing (DHH; Bavelier et al., 2006). Without early identification, technology use, and auditory-based intervention, hearing loss is often accompanied by difficulty developing spoken language (Taitelbaum-Swead et al., 2006), and it may also bring about disadvantages in daily life and societal discrimination (Branson and Miller, 2005). However, several studies have also suggested that the removal of one sensory modality leads to neural reorganization of the remaining modalities (Finney et al., 2001), which is regarded as compensation.

It is well established that gestures are communicative and are integrated automatically with speech by individuals with typical hearing in order to comprehend a message (Volterra and Erting, 1994; Obermeier et al., 2011, 2012). Language researchers have theorized that gesture and speech work together to form a single integrated system of meaning during language comprehension (Kendon, 1986; McNeill, 1994; Hostetter, 2011; Dargue et al., 2019; Kandana Arachchige et al., 2021). Kelly et al. (1999) pioneered research into what they termed the gesture–speech integration (GSI) effect and argued that gestures have a powerful impact on how hearing individuals comprehend and remember pragmatic communication. This automatic GSI also appears to exist in DHH individuals. Obermeier et al. (2012) conducted two experiments to investigate automaticity and the ways in which communicative abilities and the environment influence integration of gesture and speech. They found a significant benefit of using gestures during communication among the DHH group; that is, DHH participants showed better GSI and successful disambiguation of speech. Results indicated that gestures are beneficial in countering difficult communication conditions independent of whether the difficulties are due to external (ambient noise) or internal (hearing loss) factors.

### Representational Gestures and Spoken Language Comprehension

In general, representational gestures, which convey meaning *via* their form and motion, facilitate comprehension of both native and non-native languages (Rogers, 1978; Beattie and Shovelton, 1999; Kelly et al., 1999, 2010b; Church et al., 2004; Holle and Gunter, 2007; Holler and Wilkin, 2011; Obermeier et al., 2011). These gestures help to disambiguate pragmatically ambiguous speech (Kelly et al., 1999) and words with ambiguous meanings (Holle and Gunter, 2007). Moreover, semantic processing is impacted by the presence of representational gestures either congruent or incongruent in meaning with co-occurring speech (Kelly et al., 2004). Moreover, they play a crucial role in language-based communication (Kelly et al., 2010b), and people extract information about meaning from them (Beattie and Shovelton, 1999). Representational gestures are taken into consideration during conversations, and one cannot avoid integrating them with speech (Kelly et al., 2010a). Representational gestures improve speech comprehension in suboptimal situations, such as in a setting with a great deal of background noise, influencing GSI (Obermeier et al., 2011; Drijvers and Özyürek, 2017). In these situations, individuals tend to regard representational gestures as a helpful and related cue for language comprehension (Rogers, 1978). Overall, representational gestures have profound influences on the processing, communication, and comprehension of spoken language.

### Gesture and Speech Comprehension in the Typically Hearing Population

As discussed above, gesture, as a non-verbal disambiguation cue, influences speech interpretation among individuals with typical hearing. In particular, gestures are a relevant and helpful cue in noisy conversational settings (Rogers, 1978). According to Obermeier et al. (2011), typically hearing individuals leverage gestures to disambiguate the meaning of ambiguous speech when noise interferes with its comprehension. In the field of embodied cognition, iconic gestures, which represent physical/spatial attributes or actions, have been widely studied because of their direct connection to tangible conceptual representations (Barsalou, 2003). McNeil et al. (2000) have investigated whether these gestures support spoken language comprehension in children. They conclude that their role in speech comprehension depends on the complexity of the spoken message, and that they facilitate speech comprehension primarily for complex spoken messages. In typically hearing individuals, speech and gesture reciprocally influence one another’s semantic processing during online comprehension (Ozyurek, 2010). Gesture has also been found to influence three interrelated cognitive processes sub-serving second language (L2) word learning: communication, encoding, and recall (Allen, 1995; Tellier, 2008; Macedonia et al., 2011; Macedonia and von Kriegstein, 2012; So et al., 2012; Macedonia, 2014; Morett, 2014, 2018). Above all, the effect of gesture on speech comprehension in hearing individuals depends on both the relation of gesture to speech and the complexity of the spoken message (McNeil et al., 2000), facilitating language processing and communication among native and non-native speakers.

Several studies suggest that gestures complementing spoken language in meaning facilitate learning (Sadoski, 2018; Andra et al., 2020). Porter (2016) and Tellier (2008) examined the effects of gesture production on children’s L2 vocabulary acquisition. Tellier (2008) demonstrated that production of gestures conveying the meanings of L2 words may facilitate children’s L2 vocabulary learning, whereas Porter (2016) shows that such gestures may facilitate it for 5- and 6-year-old when combined with images of referents. Andra et al. (2020) compared the effect of gestures and images depicting word referents on children’s L2 vocabulary learning. They conclude that gesture significantly benefits L2 vocabulary learning in comparison with learning without gesture, whereas it does not significantly benefit it in comparison with images. Further, the effects of gesture on L2 vocabulary learning last for several months, indicating that it facilitates long-term memory for L2 words.

Gestures also facilitate the understanding of abstract concepts, and different types of gestures have different effects on it (Kang et al., 2013). Moreover, gestures benefit comprehension of spoken narratives (Hough, 1990; Lyle, 2000; Schmithorst et al., 2006; Dargue and Sweller, 2020). Finally, there is growing evidence that gestures can enhance acquisition of novel L2 speech sounds (Morett and Chang, 2015; Zheng et al., 2018; Baills et al., 2019; Zhen et al., 2019; Hoetjes and Van Maastricht, 2020; Xi et al., 2020; Morett et al., 2022).

### Gesture Comprehension and Use in the DHH Population

Many representational gestures approximate signs from signed languages, which may aid communication between individuals who are DHH and those with typical hearing. Kendon (1997) considered gesticulations (i.e., co-speech gestures), emblems, and signs all to be gestures, but did not consider posture shifts, self-adaptors (e.g., grooming, scratching), and object manipulations to be gestures. It is likely that DHH individuals have permanently adapted their communicative systems to incorporate as much extra-linguistic information as possible (Obermeier et al., 2012). Deaf people often fixate visually on the face to pick up microexpressions and movements of the articulators (Muir and Richardson, 2005). Thus, individuals experiencing difficulties hearing speech tend to use available visual information to improve their speech comprehension (Muir and Richardson, 2005). This makes co-speech gestures a powerful tool to support speech comprehension in daily communication.

Comprehension of co-speech gestures has been researched more extensively in DHH individuals than comprehension of other gesture types because of their supporting role in language processing (Krauss et al., 1991; McNeill, 1994; Kelly et al., 2010b). In oral-deaf individuals who tend to be born to hearing parents and learn to communicate orally and to read words on the lips of the speakers (Vendrame et al., 2010), gestures accompanying discourse facilitate retention of content information and correct inferences. However, co-speech gestures interfere with verbatim memory for discourse in these individuals. In addition, gestures produced by DHH individuals also complement sign language; for example, gestures can be used to request a turn during a sign language conversation (Emmorey, 1999).

### GSI in the Hearing and DHH Populations

GSI is an automatic process supporting language comprehension. When a semantically incongruent gesture–speech combination is presented, processing of gesture is negatively affected by incongruent speech, and processing of speech is also negatively affected by incongruent gesture. That is, concurrent speech and gestures influence each other’s processing (Kelly et al., 2010b). Kelly et al.’s (1999) seminal work proposed the GSI effect and argued that gestures have a powerful impact on how speech is comprehended and remembered. Kelly et al. (2010b) further explored the strength of the neural relationship between gesture and speech by examining a potential interface between language and action in the brain (i.e., GSI). Through a Stroop-like task, this study provided evidence supporting the GSI effect. When participants’ attention was drawn to the semantic relationship between speech and gesture, a larger N400 effect (which indexes semantic integration, as in Kutas and Hillyard, 1980) was observed when spoken words were accompanied by semantically incongruent vs. congruent gestures. Zhao et al. (2018) explored the neurocognitive control mechanism of GSI using transcranial magnetic stimulation (TMS), showing that disrupting activity in related brain regions (inferior frontal gyrus or posterior middle temporal gyrus) selectively impairs GSI.

Language comprehension is also influenced by gesture in GSI tasks in DHH individuals (Obermeier et al., 2011, 2012). Obermeier et al.’ (2012) seminal study examined the GSI effect in DHH individuals to determine whether gestures influence their comprehension to a greater extent than that of hearing individuals. They found that spoken language comprehension in DHH individuals is heavily influenced by gesture, like typically hearing subjects in the noisy condition. It seems that individuals with normal hearing adapt their gesture production and comprehension based on the quality of the auditory speech signal, whereas DHH individuals have permanently adapted their communication to incorporate as much extra-linguistic information as possible, leading them to incorporate gesture with spoken language with greater automaticity.

### Cross-Modal Plasticity and Multimodal Integration in the DHH Population

DHH and hearing individuals differ in visual cognitive ability, spatial distribution of attention to the peripheral field, and multimodal reorganization. Enhancements in visual cognition have been noted in DHH individuals in comparison with hearing individuals when confounding variables are controlled. These changes are limited to aspects of vision that are attentionally demanding and benefit from auditory–visual convergence (Bavelier et al., 2006). Moreover, deafness appears to shift the spatial distribution of attention such that attention to the peripheral, but not the central, visual field is heightened (Bavelier et al., 2000, 2001, 2006; Obermeier et al., 2012). When asked to detect the direction of motion of a peripherally located stimulus, deaf individuals do so more quickly and accurately than hearing individuals (Neville and Lawson, 1987). Furthermore, effective connectivity between middle temporal (MT)/middle superior temporal (MST) and posterior parietal cortex is stronger in deaf than hearing individuals during peripheral but not central attention. Greater sensitivity to peripheral motion enables deaf individuals to process large, swift hand movements like signs and gestures efficiently even when focusing attention on the interlocutor’s face (Muir and Richardson, 2005). Thus, hearing loss may, to some extent, facilitate visual skills in DHH individuals compared with hearing individuals. Visual stimuli activate the auditory cortex in deaf individuals who sign, suggesting that the removal of one sensory modality in humans leads to neural reorganization of the remaining modalities, at least for those who use signed language (Finney et al., 2001). A common feature of functionally reorganized brain areas in DHH individuals is their role in multimodal processing, reinforcing recent views on the importance of multimodal integration at all stages of cognitive processing (Ghazanfar and Schroeder, 2006). Overall, these results suggest that cross-modal plasticity may serve as a core compensatory mechanism *via* enhanced modulation of spatial attention in the visual modality (Eimer et al., 2002).

### Present Study and Hypotheses

Previous research has demonstrated that language is linked to action *via* gesture (Willems, 2007; Willems and Hagoort, 2007; Hostetter and Alibali, 2010). The present study investigates the strength of this relationship and extends it to adolescents by focusing on a potential interface between two systems: representational gestures and speech.

*First*, considering evidence that gesture is similarly semantically related to speech and text (Hughes-Berheim et al., 2020), we compared the GSI effect across the visual and auditory modalities to examine how gesture is integrated with language in both modalities.

*Second*, we investigated the automaticity of GSI by using a modified Stroop task in which participants were asked to identify the speaker’s gender (a superficial task) to decrease attention on semantic congruency or incongruency between the prime and target (a goal task), following Kelly et al. (2010a).

*Third*, the GSI effect was compared in DHH and hearing adolescents to explore whether DHH individuals experience greater automaticity in integrating gestures and speech than the typical hearing group.

We hence hypothesized that (1) the GSI effect would not show a significant difference between auditory and visual modalities for either the DHH or the hearing group due to the DHH group’s use of assistive hearing technology (i.e., hearing aids or cochlear implants); (2) both DHH and hearing adolescents would respond slower to gestures and speech when they were incongruent compared to when they were congruent; and (3) the GSI effect would be stronger in DHH than hearing adolescents indicating DHH individuals experience greater automaticity in integrating gestures and speech than typical hearing individuals. Because the experimental conditions for both groups were identical, consisting of acoustically and visually clear recordings, this should eliminate the potentially confounding effects of background noise and other distractions, allowing any differences between the DHH and hearing groups to be attributed to differences in their processing of gestures accompanying spoken and written language.

## Materials and Methods

### Participants

Thirty-eight native speakers of Chinese provided written informed consent to participate in the current study. All participants were all right-handed, had normal or corrected-to-normal vision, had no known neurological deficits, and had not taken part in a similar experiment using the same stimuli. Nineteen participants (10 females; *M_age_* = 13 years, age range: 11–15 years) were DHH. We recruited adolescents as the target population because relatively little research has been conducted on GSI in adolescents (Dargue et al., 2019). The hearing and DHH groups were age matched. Seventy percent of the DHH participants had mild-to-moderate hearing loss (unaided pure tone average range: 70–115 dB); the other 30% had severe to profound hearing loss (unaided pure tone average range <70 dB; Baille et al., 1996). Most DHH participants used sign language in daily communication. Only two participants reported that they used spoken language in their interactions with the general population. DHH participants were recruited from educational programs where spoken Chinese and Chinese Sign Language were used simultaneously by teachers and students throughout the day. All DHH participants communicated in spoken Chinese during the experiment, and all used assistive hearing technology (i.e., hearing aids or cochlear implants) to access speech sounds during the experiment. Eighty-five percent of the DHH participants had hearing parents. Nineteen participants (nine females; *Mean_age_* = 13.45 years, age range: 13–18 years) had typical hearing. Hearing levels of participants in the control group were tested using an ISO-audiogram with the frequency bands 500, 1,000, 2,000, and 4,000 Hz (Moore, 2014). They had a mean hearing level of 13 dB (range: 6–19 dB), which is well within the 25 dB range typically defined as the boundary for normal hearing (Bies and Hansen, 2003). All participants were paid $10 USD for their participation.

### Materials

**Figure 1:**
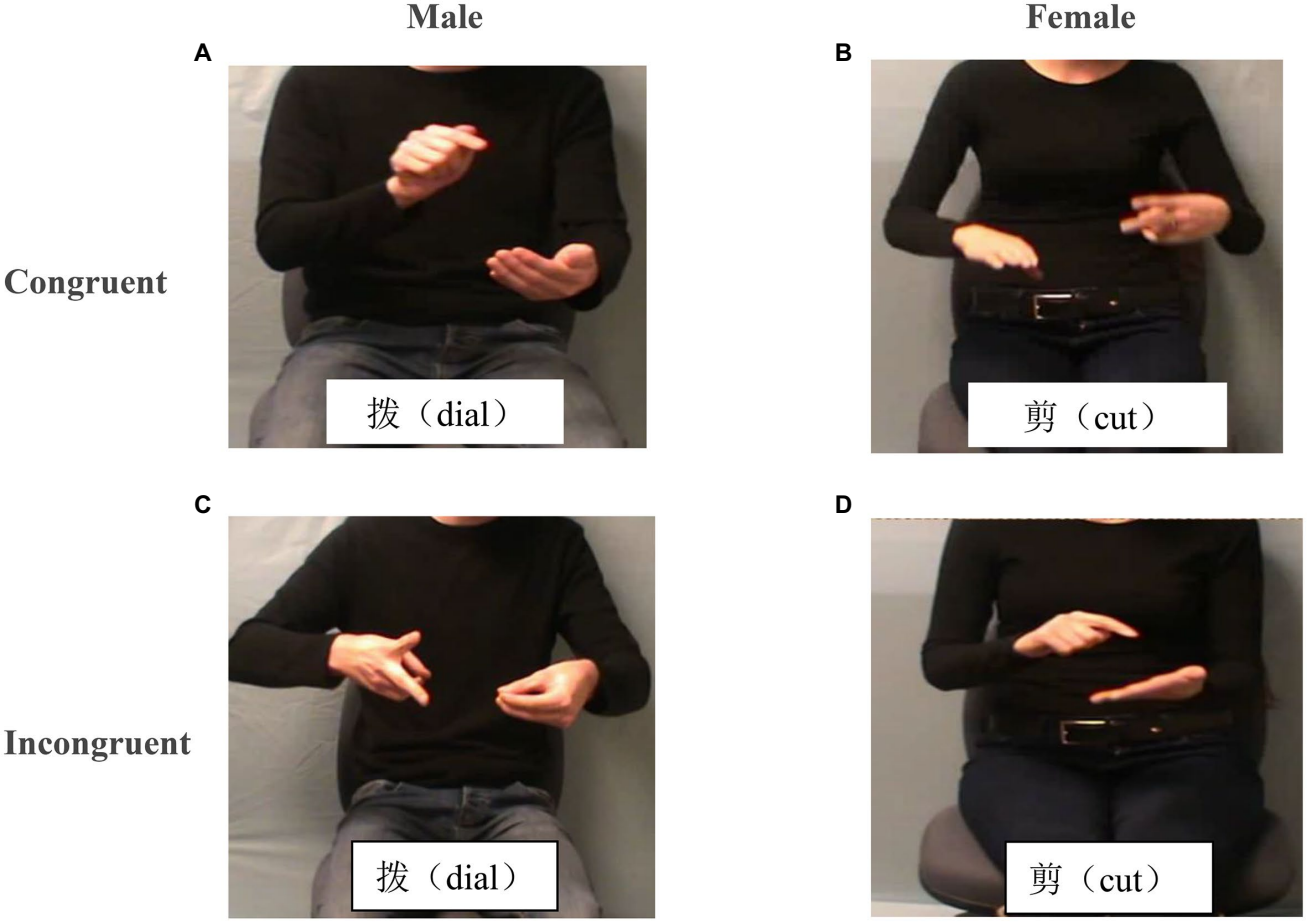
Semantically congruent and incongruent Stroop-like gesture–speech integration (GSI) task paradigm. Illustrations of the stimuli. In the gesture-congruent condition (picture **A**, **B**), the gesture and the speech were semantically related (e.g., gesturing dial and saying “*dial*” or gesturing cut and saying “*cut*”), and in the gesture-incongruent condition (picture **C**, **D**), they were semantically unrelated (e.g., gesturing cut and saying “*dial*,” and vice versa). There was a gender manipulation that varied the relationship between the gesturer and the speaker. In the gender-same condition, the gesturer and the speaker were the same person (a man or a woman), but in the gender-different condition, the gesturer and the speaker were two different people (e.g., a man gesturing but a woman speaking, and vice versa).

Participants viewed a randomized sequence of 120 short video clips twice (once in congruent trials, and once in incongruent trials). A semantically related or unrelated prime was shown on the screen following a video clip (interleaved across trials), for a total of 240 presentations. Stimuli were divided across two groups, such that half of the trials were “related” (i.e., congruent) and the other half were “unrelated” (i.e., incongruent). For example, referring to Figure 1, suppose that the primes were the words *dial* and *cut*. Panels A and B are related (congruent), whereas Panels C and D are unrelated (incongruent), creating a completely balanced design. Participants were instructed to press one button if the gesture related to the prime and another button if it did not relate to the prime. The experimental procedure lasted approximately 20 min.

### Stimuli/Materials

Digital videos were created with a Sony DV 100 digital camcorder and edited with Final Cut Pro software. Videos showed a male or female actor situated in natural contexts (e.g., kitchen, living room, entryway) describing everyday activities (e.g., drinking water, watering, tying shoes). We used different backgrounds to increase the ecological validity of the results. The actors faced the camera in all videos, but their faces were digitally covered so that mouth movements were not visible. Subjects were told that this was to hide the actors’ identities. Actors spoke at a normal pace with no artificial pauses between words.

Forty-four digitized videos of iconic gestures (e.g., break, twist) were selected for use in the current study based on previous studies (Kelly et al., 2010a; Dick et al., 2014). Each gesture was produced by either a male or a female while simultaneously uttering the corresponding verb in Chinese (Figure 1). In a follow-up session, the two speakers were recorded producing words only as speech. Video and audio materials were then combined to create the experimental manipulations of gender and semantic congruency. The presentation software application was used to present stimuli to participants, and response buttons were counterbalanced. Half of materials required responses of “left” for male and “right” for female, and the other half were vice versa.

Prior to presentation of videos, a written Chinese word was displayed on the screen, serving as the prime in the task (see below). The word was displayed for 500 ms, followed by a blank screen for 500 ms prior to stimulus onset. Each word displayed was an action verb used in one of the experimental conditions, and it was either related or unrelated to the auditory and/or the visual information presented in the video. The variable intertrial interval between each prime-target pair ranged from 1.5 to 2.5 s at random following Kelly et al. (2010a) and Guan and Fraundorf (2020).

Prior to the experiment, participants’ classroom teachers pretaught all the vocabulary used in the task to ensure familiarity with action verbs and their meanings. This familiarity training was designed to ensure that any differences in response times were due to semantic congruency rather than receptive vocabulary knowledge (Guan et al., 2019).

### Validation of Experimental Materials and Procedures

#### Semantic Congruency Norming

To verify the semantic congruency of gesture – speech combinations, a separate set of hearing participants (*n* = 30) rated the relationship between gesture and speech in each video on a five-point Likert scale (1 = no relation, 5 = very strong relation). The mean rating for congruent videos was 4.82 (*SD* = 0.41), whereas the mean rating for the incongruent videos was 1.21 (*SD* = 0.28), differing significantly between groups (*t* = 5.11, *p* < 0.001).

#### Validation of Paradigm and Stimulus Set

The RT paradigm by which participants indicated whether the stimuli were congruent or incongruent was validated in hearing participants with similar language backgrounds and abilities. The findings of Kelly et al. (2010a) were replicated, validating the stimulus set and procedures in both the DHH and the typical hearing groups.

### Procedures

We used a Stroop-like paradigm (Kelly et al., 2010a) to test GSI. The classic Stroop technique presents color words in different colored fonts, and the Stroop effect arises when the meaning of the written word influences how quickly and accurately the color of the font can be named (Stroop, 1935). We used a modified version of the classic Stroop procedure in which we asked participants to judge the gender of the voice of the speaker in the video as a superficial task to avoid explicitly drawing attention to gesture and speech, which may unintentionally encourage conscious and strategic processing of the two modalities. The gender congruency task was also examined to explore the automaticity of GSI effect. Because standard Chinese was used in all stimuli, dialectical differences should not have influenced gesture processing.

In the modified Stroop task, participants responded as quickly and as accurately as possible by pressing a button to indicate whether the voice in the video was a male or a female. Each video started with the onset of a gesture stroke, with speech onset occurring 200 ms later. Practice trials were provided to ensure that all participants reached 100% accuracy on gender judgments. Accuracy was at ceiling in this task; therefore, it was not analyzed. The 7.1% of trials with errors in gender judgment were excluded from RT analyses. RTs were calculated relative to spoken word onset. Outliers were defined as RTs 2.5 or more SDs outside of each individual participant’s mean RT. Overall, this resulted in 8.2% of trials being excluded as outliers, within the 5%–10% region recommended by Ratcliff (1993).

### Design and Analyses

A 2 (modality, auditory vs. visual) x 2 (semantic congruency, congruent vs. incongruent) x 2 (gender congruency, congruent vs. incongruent) x 2 (group, DHH vs. hearing/control) repeated-measures ANOVA was conducted with modality, semantic congruency, and gender congruency as within-participant factors, group as a between-participant factor, and RT as a dependent variable.

Above all, we assessed the effect of modality on GSI across groups. Then, we further assessed the overall GSI effect by collapsing across groups. To manipulate gender congruency, the genders of the voice and the actor in gesture videos were counterbalanced to either match or mismatch. This is a key characteristic of the Stroop-like task introduced by Kelly et al. (2010a). Therefore, the first step of analyses was conducted to reveal the automaticity of GSI by assessing the gender congruency effect. To manipulate semantic congruency, a gesture was paired with a semantically incongruent speech token (e.g., gesturing ironing while saying “whisk”). Importantly, the reverse combination was also presented (e.g., gesturing whisking while saying “iron”), ensuring that item-specific effects were counterbalanced in modality across the stimulus set. The goal of the experiment was to test sensitivity to semantic congruency, but the superficial task requirement was to indicate whether the voice of the speaker was male or female by pressing the corresponding button.

When interactions between congruency and group reached significance, we examined GSI effects separately in the DHH and typical hearing groups, and we conducted planned orthogonal *t*-tests (two-tailed) to determine the effect sizes for audio and visual target stimuli.

## Results

We excluded all incorrect and skipped trials from the data, as well as outliers (±2.5 SD). First, a 2 × 2 × 2 × 2 repeated-measures ANOVA performed on RT data, with semantic congruency (congruent, incongruent), gender congruency (congruent, incongruent), modality (auditory, visual) as within-participant factor and group (DHH, control) as between-participant factor, was conducted to examine the main effect of modality and modality x semantic congruency x gender congruency interaction effect. In order to answer the three research questions, we would first examine the main effect of modality and the interaction effects involved with the modality to test the first null hypothesis of modality. Then we tested the second hypothesis of the overall GSI effect by examining the interaction effects of semantic congruency by gender congruency in both groups. Finally, to test the group difference hypothesis, we conducted the simple main effects between groups (typical hearing vs. DHH).

A 2 (modality, auditory and visual) x 2 (semantic congruency, congruent and incongruent) x 2 (gender congruency, congruent and incongruent) x 2 (group, DHH and hearing/control) repeated-measures ANOVA was performed on RT data using semantic congruency, gender congruency, and modality as within-participant factors and group as a between-participant factor. There was no significant main effect for modality [*F* (1,38) = 2.151, *p* = 0.131, *ƞ*^2^ = 0.03] and no significant modality x gender congruency x semantic congruency interaction effect [*F* (1,38) = 2.177, *p* = 0.108, *ƞ*^2^ = 0.03], indicating that the effect of gender congruency does not vary by modality. Table 1 shows the RTs across the gender and semantic conditions in two modalities across groups. There were no differences in effect size in the overall GSI effect between audio vs. visual target stimuli. See the marginal means between the two modalities in Table 1.

**Table 1:** RTs across the gender and semantic conditions in two modalities across groups.

	Audio		Visual		Marginal mean
	SC	SI	SC	SI	
Gender same	621 (98)	660 (86)	614 (92)	647 (101)	636
Gender different	652 (102)	678 (98)	636 (101)	650 (102)	656
Marginal mean	635	659	624	648	----

sc, semantic congruent and si, semantic incongruent. SDs were presented in the parenthesis.

To test the overall GSI effect, the 2 (semantic congruency, congruent and incongruent) x 2 (gender congruency, congruent and incongruent) x 2 (group, DHH and hearing/control) repeated-measures ANOVA performed on RT data revealed the main effect of gender congruency reached significance, *F* (1,38) = 37.271, *p* < 0.001, *ƞ*^2^ = 0.08. RTs were longer when the gender of the spoken voice and the speaker in the video were incongruent (*M* = 656 ms, *SE* = 15 ms) compared to when they were congruent (*M* = 638 ms, *SE* = 16 ms); and the simple effect of semantic congruency is significant, *F*(1,39) = 5.327, *p* = 0.026, *ƞ*^2^ = 0.11, indicating longer RTs when gesture and speech were incongruent (*M* = 650 ms, *SE* = 15 ms) compared to when they were congruent (*M* = 630 ms, *SE* = 15 ms).

**Figure 2:**
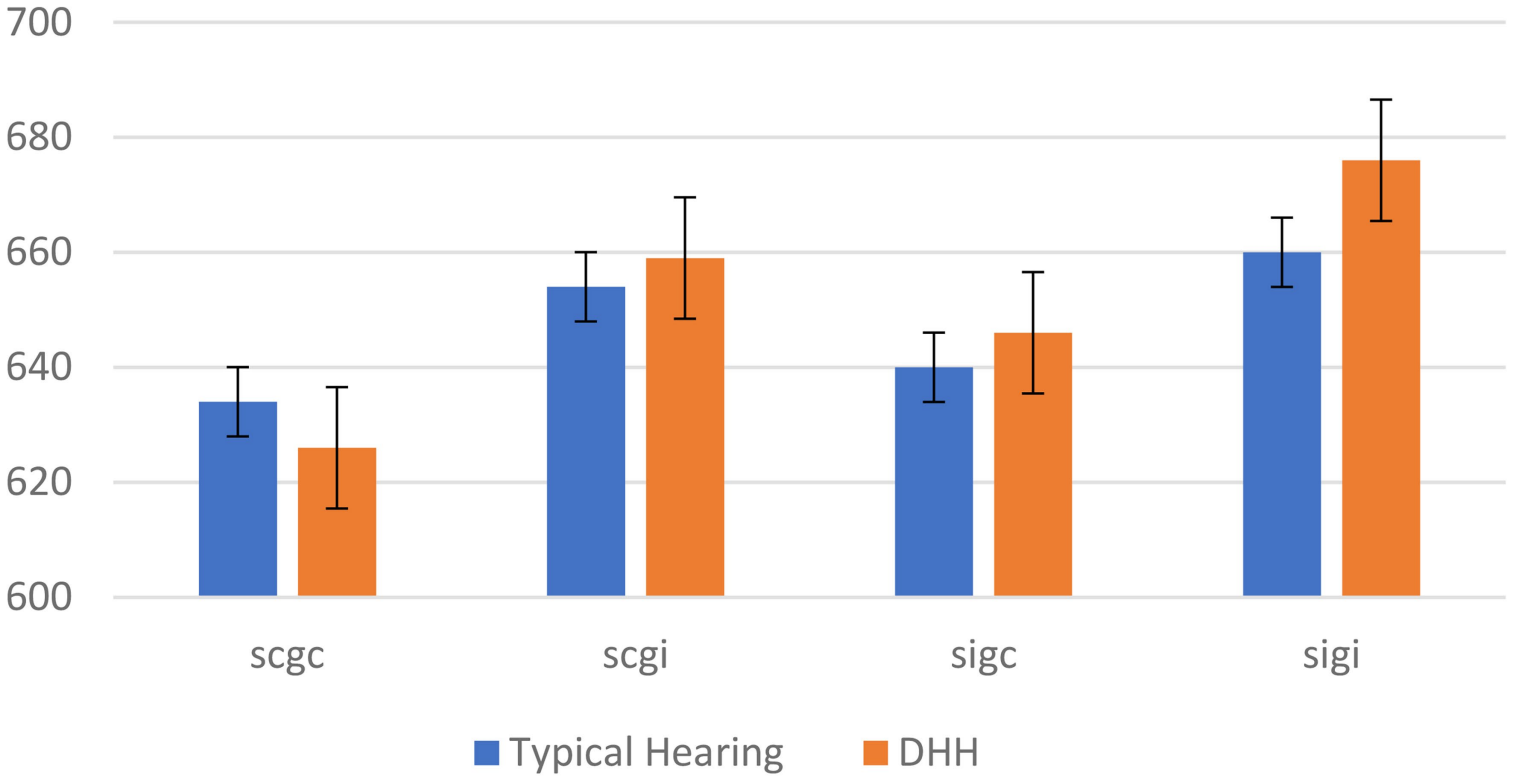
RTs for semantic congruency and gender congruency between groups. scgc, semantic congruent and gender congruent; scgi, semantic congruent and gender incongruent; sigc, semantic incongruent and gender congruent; and sigi, semantic incongruent and gender incongruent.

Simple main effects revealed that, for the between-participant group factor (typical hearing vs. DHH), there was a significant effect of gender congruency (*F* (1,38) = 38.12, *p* < 0.001, *ƞ*^2^ = 0.17), and a significant effect of semantic congruency, *F* (1,38) = 46.17, *p* < 0.001, *ƞ*^2^ = 0.22. Specifically, in the DHH group, RTs were longer when the gender of the spoken voice and the individual in the video were incongruent (*M* = 658 ms, *SE* = 12 ms) compared to when they were congruent (*M* = 625 ms, *SE* = 12 ms) with a marginal means around 33 ms, whereas this was not the case in the control group (*M* = 651 ms, *SE* = 12 ms for incongruent trials, and *M* = 634 ms, *SE* = 12 ms for congruent, with a difference around 17 ms). Moreover, in the DHH group, RTs were longer when gestures and speech were incongruent (*M* = 639 ms, *SE* = 12 ms) compared to when they were congruent (*M* = 585 ms, *SE* = 12 ms) with a difference around 64 ms, whereas this was not the case in the typical hearing control group (*M* = 630 ms, *SE* = 12 ms for incongruent trials, and *M* = 592 ms, *SE* = 12 ms for congruent, with a difference around 38 ms). Figure 2 shows the marginal means of these simple effects on RTs for semantic congruency and gender congruency by group. All significance levels were smaller than 0.05.

## Discussion

In the current study, we examined whether GSI differed between hearing and DHH adolescents to investigate how it relates to reading. Using a Stroop-like lexical decision task with visually presented Chinese characters (e.g., 剪 “cut” or 拨 “dial”) with speech consistent or inconsistent with the meanings of gestures, participants were asked to decide whether speakers were male or female. Responses in this task indicated high levels of automaticity among all participants, showing nearly 100% accuracy, but varied in RT between conditions. Three major findings were obtained. First, we found GSI in both DHH and hearing participants did not differ in the visual and auditory modalities, which are both important for language processing. Second, we found that automaticity in GSI among DHH participants differed by semantic and gender congruency in both the audio and visual conditions. Third, a comparison between DHH and hearing adolescents suggested differences in the magnitude of semantic and gender congruency effects between the two groups. There were significantly larger effects of semantic congruency and gender congruency in DHH participants compared to hearing participants, with a greater difference for semantic than for gender congruency. In other words, incongruency of the visual and auditory modalities more negatively influenced GSI among hearing participants in comparison with DHH participants. Thus, modality may not influence language processing in DHH individuals to the extent that it does in hearing individuals. To conclude, GSI in the DHH individuals is not restricted by modality, at least in our experimental task.

### Cross-Modal Plasticity of GSI Among DHH Individuals

Our study is one of few empirical studies establishing the GSI effect in DHH and hearing individuals. We not only revealed automaticity of GSI among DHH individuals, but also found that it was unaffected by modality. Previous research suggests that when the brain is deprived of input from one sensory modality, this loss is often compensated in one or more intact sensory systems. In this way, DHH individuals may compensate for decreased auditory input through visual processing, and this compensation may contribute to GSI automaticity. For instance, for DHH individuals who do not experience acoustic input, it has been shown that cross-modal reorganization of auditory cortex might provide compensatory visual function (Lomber et al., 2010). In other words, individuals with hearing loss are more likely to take advantage of available visual information and to regard this information as a default strategy to enhance their speech understanding.

DHH individuals recruit a special brain region, the motion processing area in middle temporal lobe, during peripheral attention after deafness (Bavelier et al., 2001). Meanwhile, DHH signers show increased activation of the posterior parietal cortex, supporting the view that parietal functions are modified after early auditory deprivation. Bavelier et al. (2001) studied the impact of early auditory deprivation on the organization of neural systems for visual motion processing and suggested that the polymodal area was modified after early sensory deprivation. This polymodal area refers to the dorsal “where” visual pathway projecting to the parietal cortex and is specialized for the perception of motion and for the localization of objects (Bavelier et al., 2001).

Our results provide evidence that visual attention in DHH individuals is comparable to that of hearing individuals. This finding is supported by Bavelier et al. (2000), who compared congenitally deaf and hearing individuals’ monitoring of moving stimuli occurring in the center of the visual field and found that deaf individuals devote more attention to peripheral visual space. Bavelier et al. (2006) explored whether DHH individuals had better visual skills, observing enhanced visual cognition in this population. Importantly, auditory deprivation was associated with enhanced peripheral, compared with central, visual attention. Furthermore, Finney et al. (2001) illustrated that visual stimuli were processed in the auditory cortex in deaf individuals, providing evidence that impoverished auditory input brings about neural reorganization of visual processing. Similarly, Proksch and Bavelier (2002) demonstrated that deaf individuals attended more to the visual periphery and less to the center compared to hearing individuals.

### Automaticity of GSI in DHH Individuals

Participants were slower to judge the gender of the speaker when gesture and speech were semantically incongruent, even though the semantic relationship between gesture and speech was not relevant to the task. The RT cost incurred by semantically incongruent gesture–speech pairs suggests that the representational content of gesture is automatically integrated with the representational content of speech. However, incongruent gesture–speech combinations elicited larger reaction time costs in DHH participants as compared to hearing participants, suggesting greater automaticity of GSI in DHH participants.

Some might predict that DHH individuals cannot distinguish differences in semantic congruence in gesture–word pairs when presented with gestures and semantically matching or mismatching Chinese characters. However, the results of the current study demonstrate that an automatic GSI effect exists among DHH adolescents. Even though the magnitude of the GSI effect was not as large in hearing participants as in DHH, the difference in the GSI effect between the two groups was marginal. The GSI effect in DHH individuals indicates that they integrate the semantics of gestural cues with speech.

This finding is consistent with previous research examining cognitive strategies that DHH individuals use to improve speech comprehension. For instance, Obermeier et al. (2012) found that DHH participants compensated for hearing loss by incorporating as many gestural cues as possible to improve speech comprehension. Our experimental stimuli were inspired by the stimuli of Holle and Gunter (2007), gesture fragments from Grosjean (1996), and multi-speaker babble speech created by overplaying speech streams used by Kelly et al. (2010a). Based on our behavioral data and ERP evidence from other researchers, some important conclusions can be drawn by comparing the GSI competence of DHH participants with age-matched hearing controls.

First and foremost, it appears that DHH participants tended to take gestures into account to a greater extent than hearing participants. Secondly, gestures were immediately taken into consideration by DHH participants, as their average response time in the congruent condition was quicker than in the incongruent condition. Thirdly, the accuracy rates of DHH participants in completing the experimental task were identical to hearing participants, suggesting that DHH participants may have embraced visual cues to compensate for impoverished hearing (Muir and Richardson, 2005).

For people with sensory disabilities, such as DHH, the processing of information using unaffected senses may be strengthened *via* compensation. Thus, speculatively speaking, GSI may be more efficient in DHH participants than hearing participants. In the development of hearing individuals, *skilled suppression* may be employed, resulting in slower and possibly more accurate information processing (Gernsbacher and Faust, 1995). This mechanism may have resulted in implicit GSI among hearing participants in our experiment. This conclusion requires further research, however, as we discuss in the following section.

### Limitations and Future Research

The present study has several limitations. One such limitation is the sample size. Our study included 38 participants in total, of which 19 were DHH. Further, our participants were restricted in age. We only recruited adolescents (aged 11–15), so the extent to which our results generalize to other age groups is unclear. Although our research materials were ecologically valid, the differences in natural background may have distracted participants.

There are many avenues for future research in this area. First, the cognitive processes underpinning gesture, language, and writing for DHH individuals are still underinvestigated. Guan et al. (2019), for example, researched the relations between sign language and Chinese character handwriting patterns among deaf children and revealed that their semantic priming in sign language was well-integrated with their semantic priming *via* hand writing of Chinese characters. Lexical items consisted of a finite set of hand shapes, spatial locations, and movements in sign language. Similarly, Chinese characters were made up of a finite set of radicals and forms, and these units were also spatially and visually connected to one another in writing. However, research studies on the relation between gesture and Chinese character patterns among DHH children are few and far between. Second, most studies with DHH populations have been conducted in a monolingual rather than a bilingual environment. Comparisons between DHH populations using two different written languages are rare. For instance, the question of whether differences between Chinese characters and English letters influence language processing in DHH readers remains unanswered. Third, the mechanisms used by DHH individuals during multimodal language processing remain theoretical and require more empirical study. Such research would have important implications for educators as they work to develop language (signed, spoken, or both) and literacy skills for DHH children and adolescents.

### Conclusion

A Stroop-like GSI task was used to compare the automatic GSI effect among adolescents who are DHH and those with typical hearing. Results suggested found that automaticity in GSI among DHH participants differed by semantic congruency in both the audio and visual conditions. There were significantly larger effects of semantic congruency and gender congruency in DHH participants compared to hearing participants. Meanwhile, the incongruency of the visual and auditory modalities more negatively influenced GSI among hearing participants in comparison with DHH participants. To conclude, GSI in the DHH individuals is not restricted by modality, at least in our experimental task.

## Data Availability Statement

The original contributions presented in the study are included in the article/supplementary material; further inquiries can be directed to the corresponding author.

## Ethics Statement

The studies involving human participants were reviewed and approved by the Beijing Language and Culture University. Written informed consent to participate in this study was provided by the participants’ legal guardian/next of kin.

## Author Contributions

RY and CQG designed and conducted the study. CQG and WM analyzed the data. CQG and ERS wrote the paper. BM and LM commented and revised the paper. All authors contributed to the article and approved the submitted version.

## Funding

This study was supported by the China National Social Science Funds (#BBA180075) awarded to RY as a Principal-Investigator (PI), and CQG as a Co-PI.

## Conflict of Interest

The authors declare that the research was conducted in the absence of any commercial or financial relationships that could be construed as a potential conflict of interest.

## Publisher’s Note

All claims expressed in this article are solely those of the authors and do not necessarily represent those of their affiliated organizations, or those of the publisher, the editors and the reviewers. Any product that may be evaluated in this article, or claim that may be made by its manufacturer, is not guaranteed or endorsed by the publisher.

## References

[ref1] Allen L. Q. (1995). The effects of emblematic gestures on the development and access of mental representations of French expressions. Mod. Lang. J. 79, 521–529. doi: 10.1111/j.1540-4781.1995.tb05454.x

[ref2] Andra A. Mathias B. Schwager A. Macedonia M. von Kriegstein K. (2020). Learning foreign language vocabulary with gestures and pictures enhances vocabulary memory for several months post-learning in eight-year-old school children. Educ. Psychol. Rev. 32, 815–850. doi: 10.1007/s10648-020-09527-z

[ref3] Baille M. F. Arnaud C. Cans C. Grandjean H. du Mazaubrun C. Rumeau-Rouquette C. (1996). Prevalence, aetiology, and care of severe and profound hearing loss. Arch. Dis. Child. 75, 129–132. doi: 10.1136/adc.75.2.129, PMID: 8869193 PMC1511625

[ref4] Baills F. Suárez-González N. González-Fuente S. Prieto P. (2019). Observing and producing pitch gestures facilitates the learning of mandarin Chinese tones and words. Stud. Second. Lang. Acquis. 41, 33–58. doi: 10.1017/s0272263118000074

[ref5] Barsalou L. W. (2003). Situated simulation in the human conceptual system. Lang. Cogn. Process. 18, 513–562. doi: 10.1080/01690960344000026

[ref6] Bavelier D. Brozinsky C. Tomann A. Mitchell T. Neville H. Liu G. (2001). Impact of early deafness and early exposure to sign language on the cerebral organization for motion processing. J. Neurosci. 21, 8931–8942. doi: 10.1523/jneurosci.21-22-08931.2001, PMID: 11698604 PMC6762265

[ref7] Bavelier D. Dye M. W. G. Hauser P. C. (2006). Do deaf individuals see better? Trends Cogn. Sci. 10, 512–518. doi: 10.1016/j.tics.2006.09.006, PMID: 17015029 PMC2885708

[ref8] Bavelier D. Tomann A. Hutton C. Mitchell T. Corina D. Liu G.. (2000). Visual attention to the periphery is enhanced in congenitally deaf individuals. J. Neurosci. 20:RC93. doi: 10.1523/JNEUROSCI.20-17-j0001.2000, PMID: 10952732 PMC6772982

[ref9] Beattie G. Shovelton H. (1999). Do iconic hand gestures really contribute anything to the semantic information conveyed by speech? An experimental investigation. Semiotica 123, 1–30. doi: 10.1515/semi.1999.123.1-2.1

[ref10] Bies D. A. Hansen C. H. (2003). Engineering Noise Control: Theory and Practice. 3rd *Edn*. London, United Kingdom: Chapman and Hall.

[ref11] Branson J. Miller D. (2005). Damned for Their Difference: The Cultural Construction of Deaf People as Disabled, vol. 7. Washingtong DC: Gallaudet University Press, 129–132.

[ref12] Church R. B. Ayman-Nolley S. Mahootian S. (2004). The role of gesture in bilingual education: does gesture enhance learning? Int. J. Biling. Educ. Biling. 7, 303–319. doi: 10.1080/13670050408667815

[ref13] Colozza P. Anastasio A. R. T. (2009). Screening, diagnosing and treating deafness: the knowledge and conduct of doctors serving in neonatology and/or pediatrics in a tertiary teaching hospital. São Paulo Med. J. 127, 61–65. doi: 10.1590/s1516-31802009000200002, PMID: 19597679 PMC10964800

[ref14] Cruickshanks K. J. Wiley T. L. Tweed T. S. Klein B. E. K. Klein R. Mares-Perlman J. A.. (1998). Prevalence of hearing loss in older adults in beaver dam, Wisconsin: the epidemiology of hearing loss study. Am. J. Epidemiol. 148, 879–886. doi: 10.1093/oxfordjournals.aje.a009713 9801018

[ref15] Dargue N. Sweller N. (2020). Learning stories through gesture: gesture’s effects on child and adult narrative comprehension. Educ. Psychol. Rev. 32, 249–276. doi: 10.1007/s10648-019-09505-0

[ref16] Dargue N. Sweller N. Jones M. P. (2019). When our hands help us understand: a meta-analysis into the effects of gesture on comprehension. Psychol. Bull. 145, 765–784. doi: 10.1037/bul0000202, PMID: 31219263

[ref17] Dick A. S. Mok E. H. Raja B. A. Goldin-Meadow S. Small S. L. (2014). Frontal and temporal contributions to understanding the iconic cospeech gestures that accompany speech. Hum. Brain Mapp. 35, 900–917. doi: 10.1002/hbm.22222, PMID: 23238964 PMC3797208

[ref18] Drijvers L. Özyürek A. (2017). Visual context enhanced: the joint contribution of iconic gestures and visible speech to degraded speech comprehension. J. Speech Lang. Hear. Res. 60, 212–222. doi: 10.1044/2016_jslhr-h-16-0101, PMID: 27960196

[ref19] Eimer M. Velzen J. V. Driver J. (2002). Cross-modal interactions between audition, touch, and vision in endogenous spatial attention: ERP evidence on preparatory states and sensory modulations. J. Cogn. Neurosci. 14, 254–271. doi: 10.1162/089892902317236885, PMID: 11970790

[ref20] Emmorey K. (1999). “Do signers gesture?” in Gesture, Speech, and Sign. eds. Messing L. Campbell R. (Oxford: Oxford University Press), 133–159.

[ref21] Finney E. M. Fine I. Dobkins K. R. (2001). Visual stimuli activate auditory cortex in the deaf. Nat. Neurosci. 4, 1171–1173. doi: 10.1038/nn763, PMID: 11704763

[ref22] Gernsbacher M. A. Faust M. (1995). “Skilled suppression,” in Interference and Inhibition in Cognition. ed. Denster F. N. (London, United Kingdom: Academic Press), 295–327.

[ref23] Ghazanfar A. A. Schroeder C. E. (2006). Is neocortex essentially multisensory? Trends Cogn. Sci. 10, 278–285. doi: 10.1016/j.tics.2006.04.008, PMID: 16713325

[ref24] Grosjean F. (1996). Gating. Lang. Cogn. Process. 11, 597–604. doi: 10.1080/016909696386999

[ref25] Guan C. Q. Fraundorf S. H. (2020). Cross-linguistic word recognition development among Chinese children: a multilevel linear mixed-effects modeling approach. Front. Psychol. 11:544. doi: 10.3389/fpsyg.2020.00544, PMID: 32373000 PMC7176983

[ref26] Guan C. Q. Zhao J. Kwok R. K. W. Wang Y. (2019). How does morphosyntactic skill contribute to different genres of Chinese writing from grades 3 to 6? J. Res. Read. 42, 239–267. doi: 10.1111/1467-9817.12239

[ref27] Hoetjes M. Van Maastricht L. (2020). Using gesture to facilitate L2 phoneme acquisition: the importance of gesture and phoneme complexity. Front. Psychol. 11:575032. doi: 10.3389/fpsyg.2020.575032, PMID: 33329219 PMC7719629

[ref28] Holle H. Gunter T. C. (2007). The role of iconic gestures in speech disambiguation: ERP evidence. J. Cogn. Neurosci. 19, 1175–1192. doi: 10.1162/jocn.2007.19.7.1175, PMID: 17583993

[ref29] Holler J. Wilkin K. (2011). Co-speech gesture mimicry in the process of collaborative referring during face-to-face dialogue. J. Nonverbal Behav. 35, 133–153. doi: 10.1007/s10919-011-0105-6

[ref30] Hostetter A. B. (2011). When do gestures communicate? A meta-analysis. Psychol. Bull. 137, 297–315. doi: 10.1037/a0022128, PMID: 21355631

[ref31] Hostetter A. B. Alibali M. Q. (2010). Language, gesture, action! A test of the gesture as simulated action framework. J. Mem. Lang. 63, 245–257. doi: 10.1016/j.jml.2010.04.003

[ref32] Hough M. S. (1990). Narrative comprehension in adults with right and left hemisphere brain-damage: theme organization. Brain Lang. 38, 253–277. doi: 10.1016/0093-934x(90)90114-v, PMID: 1691038

[ref33] Hughes-Berheim S. S. Morett L. M. Bulger R. (2020). Semantic relationships between representational gestures and their lexical affiliates are evaluated similarly for speech and text. Front. Psychol. 11:2808. doi: 10.3389/fpsyg.2020.575991, PMID: 33192884 PMC7642993

[ref34] Kandana Arachchige K. G. Simoes Loureiro I. Blekic W. Rossignol M. Lefebvre L. (2021). The role of iconic gestures in speech comprehension: an overview of various methodologies. Front. Psychol. 12:634074. doi: 10.3389/fpsyg.2021.634074, PMID: 33995189 PMC8118122

[ref35] Kang A. Hallman G. L. Son L. K. Black J. B. (2013). The different benefits from different gestures in understanding a concept. J. Sci. Educ. Technol. 22, 825–837. doi: 10.1007/s10956-012-9433-5

[ref36] Kelly S. D. Barr D. Church R. B. Lynch K. (1999). Offering a hand to pragmatic understanding: the role of speech and gesture in comprehension and memory. J. Mem. Lang. 40, 577–592. doi: 10.1006/jmla.1999.2634

[ref37] Kelly S. D. Creigh P. Bartolotti J. (2010a). Integrating speech and iconic gestures in a Stroop-like task: evidence for automatic processing. J. Cogn. Neurosci. 22, 683–694. doi: 10.1162/jocn.2009.21254, PMID: 19413483

[ref38] Kelly S. D. Kravitz C. Hopkins M. (2004). Neural correlates of bimodal speech and gesture comprehension. Brain Lang. 89, 253–260. doi: 10.1016/s0093-934x(03)00335-3, PMID: 15010257

[ref39] Kelly S. D. Özyürek A. Maris E. (2010b). Two sides of the same coin: speech and gesture mutually interact to enhance comprehension. Psychol. Sci. 21, 260–267. doi: 10.1177/0956797609357327 20424055

[ref40] Kendon A. (1986). Some reasons for studying gesture. Semiotica 62, 3–28. doi: 10.1515/semi.1986.62.1-2.3

[ref41] Kendon A. (1997). Gesture. Annu. Rev. Anthropol. 26, 109–128. doi: 10.1146/annurev.anthro.26.1.109

[ref42] Krauss R. M. Morrel-Samuels P. Colasante C. (1991). Do conversational hand gestures communicate? J. Pers. Soc. Psychol. 61, 743–754. doi: 10.1037/0022-3514.61.5.743, PMID: 1753329

[ref43] Kutas M. Hillyard S. A. (1980). Reading senseless sentences: brain potentials reflect semantic incongruity. Science 207, 203–205. doi: 10.1126/science.7350657, PMID: 7350657

[ref44] Lomber S. G. Meredith M. A. Kral A. (2010). Cross-modal plasticity in specific auditory cortices underlies visual compensations in the deaf. Nat. Neurosci. 13, 1421–1427. doi: 10.1038/nn.2653, PMID: 20935644

[ref45] Lyle S. (2000). Narrative understanding: developing a theoretical context for understanding how children make meaning in classroom settings. J. Curric. Stud. 32, 45–63. doi: 10.1080/002202700182844

[ref46] Macedonia M. (2014). Bringing back the body into the mind: gestures enhance word learning in foreign language. Front. Psychol. 5:1467. doi: 10.3389/fpsyg.2014.01467, PMID: 25538671 PMC4260465

[ref47] Macedonia M. Müller K. Friederici A. D. (2011). The impact of iconic gestures on foreign language word learning and its neural substrate. Hum. Brain Mapp. 32, 982–998. doi: 10.1002/hbm.21084, PMID: 20645312 PMC6870319

[ref48] Macedonia M. von Kriegstein K. (2012). Gestures enhance foreign language learning. Biolinguistics 6, 393–416. doi: 10.5964/bioling.8931

[ref49] McNeil N. M. Alibali M. W. Evans J. L. (2000). The role of gesture in children's comprehension of spoken language: now they need it, now they don't. J. Nonverbal Behav. 24, 131–150. doi: 10.1023/A:1006657929803

[ref50] McNeill D. (1994). Hand and mind: what gestures reveal about thought. Language 70, 345–350. doi: 10.2307/415833

[ref001] Moore B. C. J. (2014). Development and current status of the “Cambridge” loudness models. Trends Hear. doi: 10.1177/2331216514550620 PMC4227665 25315375

[ref51] Morett L. M. (2014). When hands speak louder than words: the role of gesture in the communication, encoding, and recall of words in a novel second language. Mod. Lang. J. 98, 834–853. doi: 10.1111/modl.12125

[ref52] Morett L. M. (2018). In hand and in mind: effects of gesture production and viewing on second language word learning. Appl. Psycholinguist. 39, 355–381. doi: 10.1017/s0142716417000388

[ref53] Morett L. M. Chang L. Y. (2015). Emphasising sound and meaning: pitch gestures enhance mandarin lexical tone acquisition. Lang. Cogn. Neurosci. 30, 347–353. doi: 10.1080/23273798.2014.923105

[ref54] Morett L. M. Feiler J. B. Getz L. M. (2022). Elucidating the influences of embodiment and conceptual metaphor on lexical and non-speech tone learning. Cognition 222:105014. doi: 10.1016/j.cognition.2022.105014, PMID: 35033864

[ref55] Moscicki E. K. Elkins E. F. Baum H. McNarnara P. M. (1985). Hearing loss in the elderly: an epidemiologic study of the Framingham heart study cohort. Ear Hear. 6, 184–190. doi: 10.1097/00003446-198507000-00003 4043571

[ref56] Muir L. J. Richardson I. E. (2005). Perception of sign language and its application to visual communications for deaf people. J. Deaf. Stud. Deaf. Educ. 10, 390–401. doi: 10.1093/deafed/eni037, PMID: 16000689

[ref57] Neville H. J. Lawson D. (1987). Attention to central and peripheral visual space in a movement detection task: an event-related potential and behavioral study. II. Congenitally deaf adults. Brain Res. 405, 268–283. doi: 10.1016/0006-8993(87)90296-4, PMID: 3567605

[ref58] Obermeier C. Dolk T. Gunter T. C. (2012). The benefit of gestures during communication: evidence from hearing and hearing-impaired individuals. Cortex 48, 857–870. doi: 10.1016/j.cortex.2011.02.007, PMID: 21397223

[ref59] Obermeier C. Holle H. Gunter T. C. (2011). What iconic gesture fragments reveal about gesture-speech integration: when synchrony is lost, memory can help. J. Cogn. Neurosci. 23, 1648–1663. doi: 10.1162/jocn.2010.21498, PMID: 20350188

[ref60] Ozyurek A. (2010). “The role of iconic gestures in production and comprehension of language: evidence from brain and behavior,” in Gesture in Embodied Communication and Human-Computer Interaction. eds. Kopp S. Wachsmuth I., GW 2009. Lecture Notes in Computer Science, *Vol.* 5934 (Berlin, Heidelberg: Springer).

[ref61] Porter A. (2016). A helping hand with language learning: teaching French vocabulary with gesture. Lang. Learn. J. 44, 236–256. doi: 10.1080/09571736.2012.750681

[ref62] Proksch J. Bavelier D. (2002). Changes in the spatial distribution of visual attention after early deafness. J. Cogn. Neurosci. 14, 687–701. doi: 10.1162/08989290260138591, PMID: 12167254

[ref63] Ratcliff R. (1993). Methods for dealing with reaction time outliers. Psychol. Bull. 114, 510–532. doi: 10.1037/0033-2909.114.3.510, PMID: 8272468

[ref64] Reuben D. B. Walsh K. Moore A. A. Damesyn M. Greendate G. A. (1998). Hearing loss in community-dwelling order persons: national prevalence data and identification using simple questions. J. Am. Geriatr. Soc. 46, 1008–1011. doi: 10.1111/j.1532-5415.1998.tb02758.x, PMID: 9706892

[ref65] Rogers W. T. (1978). The contribution of kinesic illustrators toward the comprehension of verbal behavior within utterances. Hum. Commun. Res. 5, 54–62. doi: 10.1111/j.1468-2958.1978.tb00622.x

[ref66] Sadoski M. (2018). Reading comprehension is embodied: theoretical and practical considerations. Educ. Psychol. Rev. 30, 331–349. doi: 10.1007/s10648-017-9412-8

[ref67] Schmithorst V. J. Holland S. K. Plante E. (2006). Cognitive modules utilized for narrative comprehension in children: a functional magnetic resonance imaging study. NeuroIamge 29, 254–266. doi: 10.1016/j.neuroimage.2005.07.020, PMID: 16109491 PMC1357541

[ref68] So W. C. Sim Chen-Hui C. Low Wei-Shan J. (2012). Mnemonic effect of iconic gesture and beat gesture in adults and children: is meaning in gesture important for memory recall? Lang. Cogn. Process. 27, 665–681. doi: 10.1080/01690965.2011.573220

[ref69] Stroop J. R. (1935). Studies of interference in serial verbal reactions. J. Exp. Psychol. 18, 643–662. doi: 10.1037/h0054651

[ref70] Taitelbaum-Swead R. Brownstein Z. Muchnik C. Kishon-Rabin L. Kronenberg J. Megirov L.. (2006). Connexin-associated deafness and speech perception outcome of cochlear implantation. Arch. Otorhinolaryngol.- Head Neck Surg. 132, 495–500. doi: 10.1001/archotol.132.5.495, PMID: 16702564

[ref71] Tellier M. (2008). The effect of gestures on second language memorisation by young children. Gesture 8, 219–235. doi: 10.1075/gest.8.2.06tel

[ref72] Vendrame M. Cutica I. Bucciarelli M. (2010). “I see what you mean”: oral deaf individuals benefit from speaker’s gesturing. Eur. J. Cogn. Psychol. 22, 612–639. doi: 10.1080/09541440903126030

[ref73] Verrecchia B. Curcio V. (2016). Diagnosis of hearing loss in newborns and infants, through objective audiological assessment. Glob. J. Otolaryngol. 1, 85–87. doi: 10.19080/gjo.2016.01.555573

[ref74] Volterra V. Erting C. J. (1994). From Gesture to Language in Hearing and Deaf Children. Berlin: Springer-Verlag.

[ref75] Willems R. M. (2007). When language meets action: the neural integration of gesture and speech. Cereb. Cortex 17, 2322–2333. doi: 10.1093/cercor/bhl141, PMID: 17159232

[ref76] Willems R. M. Hagoort P. (2007). Neural evidence for the interplay between language, gesture, and action: a review. Brain Lang. 101, 278–289. doi: 10.1016/j.bandl.2007.03.004, PMID: 17416411

[ref77] Xi X. Li P. Baills F. Prieto P. (2020). Hand gestures facilitate the acquisition of novel phonemic contrasts when they appropriately mimic target phonetic features. J. Speech Lang. Hear. Res. 63, 3571–3585. doi: 10.1044/2020_jslhr-20-00084, PMID: 33090915

[ref78] Yuri A. Platz E. A. Niparko J. K. (2008). Prevalence of hearing loss and differences by demographic characteristics among US adults: data from the national health and nutrition examination survey, 1999-2004. Arch. Intern. Med. 168, 1522–1530. doi: 10.1001/archinte.168.14.1522, PMID: 18663164

[ref79] Zhao W. Y. Riggs K. Schindler I. Holle H. (2018). Transcranial magnetic stimulation over left inferior frontal and posterior temporal cortex disrupts gestures-speech integration. J. Neurosci. 38, 1891–1900. doi: 10.1523/jneurosci.1748-17.2017, PMID: 29358361 PMC6705889

[ref80] Zhen A. Van Hedger S. Heald S. Goldin-Meadow S. Tian X. (2019). Manual directional gestures facilitate cross-modal perceptual learning. Cognition 187, 178–187. doi: 10.1016/j.cognition.2019.03.004, PMID: 30877849

[ref81] Zheng A. Hirata Y. Kelly S. D. (2018). Exploring the effects of imitating hand gestures and head nods on L1 and L2 mandarin tone production. J. Speech Lang. Hear. Res. 61, 2179–2195. doi: 10.1044/2018_jslhr-s-17-0481, PMID: 30193334

